# Silencing lncRNA TUG1 Alleviates LPS-Induced Mouse Hepatocyte Inflammation by Targeting miR-140/TNF

**DOI:** 10.3389/fcell.2020.616416

**Published:** 2021-02-11

**Authors:** Qing-Min Liu, Li-Li Liu, Xi-Dong Li, Ping Tian, Hao Xu, Zeng-Lian Li, Li-Kun Wang

**Affiliations:** ^1^Intensive Care Unit, Linyi People’s Hospital, Linyi, China; ^2^Department of Pathology, Linyi People’s Hospital, Linyi, China; ^3^Department of Infection Control Center, Linyi People’s Hospital, Linyi, China

**Keywords:** hepatitis, lncRNA TUG1, miR-140, TNF, LPS

## Abstract

Hepatitis is a major public health problem that increases the risk of liver cirrhosis and liver cancer. Numerous studies have revealed that long non-coding RNAs (lncRNAs) exert essential function in the inflammatory response of multiple organs. Herein, we aimed to explore the effect of lncRNA TUG1 in LPS-induced hepatocyte inflammation response and further illuminate the underlying mechanisms. Mice were intraperitoneally injected with LPS, and the liver inflammation was evaluated. Microarray showed that lncRNA TUG1 was upregulated in LPS-induced hepatocyte inflammation. qRT-PCR and immunofluorescence assay indicated a significant increase of TUG1 in mice with LPS injection. Functional analysis showed that si-TUG1 inhibited LPS-induced inflammation response in mice liver, inhibited apoptosis level, and protected liver function. Then, we knock down TUG1 in normal human hepatocyte AML12. Consistent with *in vivo* results, si-TUG1 removed the injury of LPS on AML12 cells. Furthermore, TUG1 acted as a sponge of miR-140, and miR-140 directly targeted TNFα (TNF). MiR-140 or si-TNF remitted the beneficial effects of TUG1 on LPS-induced hepatocyte inflammation response both *in vitro* and *in vivo*. Our data revealed that deletion of TUG1 protected against LPS-induced hepatocyte inflammation via regulating miR-140/TNF, which might provide new insight for hepatitis treatment.

## Introduction

Hepatitis is a general term for liver inflammation, which is usually caused by various pathogenic factors, including viruses, bacteria, alcohol, drugs, and so on (Gao et al., 2020). Inflammatory injury is considered the main factor leading to liver cancer occurrence and development (Kubes and Jenne, 2018; Fan et al., 2020). Inhibition of inflammatory liver injury may be an essential strategy to control the occurrence of liver cancer (Bartneck et al., 2017). The infiltration of inflammatory cells and the production of inflammatory factors destroy the original immune balance and induce a series of pathological liver damage. At present, alanine aminotransferase and aspartate aminotransferase are used as the detection index of liver injury, which exerts essential effects in the diagnosis and treatment of liver injury. Still, the specificity is poor (Bihrer et al., 2011). Therefore, it is of great significance to explore the clinical indicators for diagnosing and treating hepatitis.

Long non-coding RNA (lncRNA) is a group of RNA molecules that are longer than 200 nt, with little or no protein-coding function (Carter et al., 2020). LncRNA plays an important role in regulating and maintaining the structural integrity of chromosome and genome, chromosome inactivation, gene transcription, translation, and epigenetic regulation (Wang et al., 2020). In recent years, studies have shown that lncRNAs can also be detected in various liquid samples and can exist in some enzymes, acids, bases, and other conditions, and still has good stability after repeated freezing and thawing (Mazar et al., 2010). The discovery of lncRNAs and the improvement of detection methods make it an ideal molecular marker and effective therapeutic target (Xi et al., 2018; Lou et al., 2019). In addition, lncRNA can be used as a new tumor diagnostic marker because of its good sensitivity and specificity (Li Z. et al., 2017b). At present, lncRNA has become a new research hotspot. Different lncRNA expressions were found by LPS stimulation in human renal tubular epithelial cells, monocytes, cardiomyocytes, or plasma of patients with sepsis (Mey et al., 2019). The differentially expressed lncRNA screened by LPS in human umbilical vein endothelial cells changed by 10 times and 72 times. These changes in lncRNAs expression may be involved in the inflammatory response. LncRNA-IL7R interacts with IL-7 receptor α subunit to reduce the pro-inflammatory response induced by LPS (Cui et al., 2014). HOTAIR and NF-κB signal pathways regulate the expression of TNFα in septic cardiomyocytes (Wu et al., 2016). LncRNA has become a key regulator of inflammation. To further understand the interaction between inflammatory signal transduction pathway and lncRNA, researchers found that cardiac and apoptosis-related Carlr is a kind of lncRNA expressed in different tissues and cells of mice and humans. After the enhancement of NF-κB signal in macrophages, the expression of Carlr increased and translocated to the cytoplasm (Castellanos-Rubio et al., 2017).

LncRNA participates in liver metabolism and capacity balance (Lan et al., 2019; Pradas-Juni et al., 2020). LncRNA-LSTR is enriched in the liver that regulates glucose and lipid metabolism in the liver. The consumption of LSTR in mouse liver upregulates the expression of ApoC2, which promotes the clearance of triglycerides (Filser et al., 2001). LncRNA taurine upregulated gene 1 (TUG1) is first identified in murine retina and is necessary for retina development (Young et al., 2005). Recent studies show that TUG1 is closely related to inflammation response in cardiomyocyte ischemia reperfusion injury and acute lung injury (Shi et al., 2019; Qiu et al., 2020). However, the role of TUG1 in hepatocyte inflammation is poorly clarified. Herein, we explored the effect of lncRNA TUG1 in LPS-induced hepatocyte inflammation and injury and further illuminate the possible underlying mechanisms.

## Materials and Methods

### Animal Experiments

C57BL/6 mice (male, about 25 g of weight) were purchased from Beijing Viton Lihua Experimental Animal Technology Company. After intraperitoneal injection of 22 mg/ml pentobarbital sodium (diluted with normal saline), the caudal root, hindlimb, and eyelash reflexes disappeared after 10 min, and slow breathing was considered as deep anesthesia. After anesthesia, the mouse head was tilted downward, and the tongue was pulled out with tweezers. One hundred fifty microliters of lentivirus (1 × 10^8^ PFU/ml) containing TUG1-shRNA/TNF-shRNA/NC-shRNA was injected in the tail vein of mice. miR-140 antagomiR (antagomiR-140)/antagomiR-NC (80 mg/kg) in 0.2 ml saline was injected once a day for 3 consecutive days. Twenty-one days after the lentivirus injection, 8 mg/kg LPS was intraperitoneally injected into mice for 6 h. Then, mice were intraperitoneally injected with 3% pentobarbital sodium and were euthanized by excessive anesthesia with a dose of 90 ml/kg, and the organs and tissues were removed for follow-up study. The research protocol of this study was approved by the Animal Care and Use Committee of the Linyi people’s hospital.

### Cell Culture and Treatment

The AML12 cell line (mouse normal hepatocytes) was purchased from the Science Cell Laboratory. Cell lines were cultured in DMEM (Thermo-Life, United States) with 10% FBS (Thermo Fisher Scientific, United States) and 100 μl/ml penicillin and streptomycin (Beyotime, China) and placed at 37°C with 5% CO_2_. The AML12 cells were plated until the cell density reached 80% confluency of dishes to transfect. AMO-140 (miR-140 inhibitor) or small interfering RNA (si-RNA) of TUG1 or TNFα (TNF) was constructed by Genechem (Shanghai, China). The plasmids were transfected with Lipofectamine 2000 (Invitrogen, Carlsbad, CA). LPS was added into culture media of cells at a concentration of 100 ng/ml for 6 h. Primary liver cells were isolated from LPS/saline-treated mice using collagenase perfusion technique as previously described (Korelova et al., 2019). Macrophages were purified from mixed primary cultures of adult mice liver cells (Kitani et al., 2011).

### MTT Assay

AML12 cells were plated in 96-well plates and we used MTT assay to detect the cell viability. MTT (0.5 mg/ml; Beyotime Biotechnology, China) was added to every well after treatment and incubated for 3 h at 37°C. One hundred microliters of DMSO was added and incubated for 15 min. We measured the absorbance by a spectrophotometer (Tecan, Austria) at 493 nm.

### ROS Assay

Reactive oxygen species (ROS) detection was performed according to the procedures (Beyotime, China). Briefly, AML12 cells were plated in 12-well plates and ROS solution was added into cells for 20 min. After fixation in 4% paraformaldehyde and PBS washing solution, the cells were incubated in DAPI for 10 min. Fluorescence was observed by fluorescence microscope.

### qRT-PCR

RNA extraction was performed using trizol reagent. NanoDrop 8000 (Thermo Fisher Scientific, Waltham, MA, United States) was used to detect the concentration and purity of RNA. The single-stranded cDNAs were synthesized from 1 μg of RNA. The expression of mRNAs and miRNAs was quantified by RT-PCR with SYBR Green I (Thermo Fisher Scientific, Inc.).

### Western Blot

After RIPA cleavage, we extracted total protein and measured it with BCA method. After quantitative denaturation, proteins were separated using 10 or 15% polyacrylamide gels and transferred onto 0.22 μm PVDF membranes (Merck Millipore, United States). The first incubation and second incubation were carried out according to the operation steps. The expression of the protein was expressed by the gray value. Primary antibodies list: GADPH (ab181602, Abcam), cleaved-caspase3 (ab2302, Abcam), Bax (ab32503, Abcam), Bcl2 (12789-1-AP, Proteintech), TNFα (17590-1-AP, Proteintech), and IL-6 (66146-1-Ig, Proteintech). The secondary antibodies IRDye700/800 Mouse or Rabbit were produced by LICOR (Lincoln, Nebraska, United States) for 1 h, and the bands were scanned using Odyssey.

### Luciferase Assay

We constructed wild-type (WT) or mutant (Mut) psiCHECK-2 luciferase reporter plasmid in GenePharma company (Shanghai, China). HEK293 cells were co-transfected with 20 mmol/L miR-140 mimic or miR-NC together with WT-TUG1/Mut-TUG1 or WT-TNF/Mut-TNF. Luciferase activity was measured with Dual Luciferase Reporter Assay Kit (Transgene, China) on GloMax20/20 at 48 h after the transfection.

### RIP

We used RIP assay to determine the binding between TUG1/TNF and miR-140 using Magna RIP^TM^ RNA-Binding Protein Immunoprecipitation Kit (Millipore) as previously reported (Liu C. et al., 2018). Briefly, AML12 cells were transfected with biotinylated miR-140/miR-NC or TNF/NC, and the mRNA level of TUG1 or miR-140 was detected using qRT-PCR.

### H&E Staining

The liver tissues were gathered and fixed in 4% paraformaldehyde for 24 h. Then, the fixed tissues were embedded in paraffin. Next, a paraffin slicer machine was used to cut slices (5 mm cross-sectional). H&E staining was used to evaluate pulmonary morphology. Liver sections were dewaxed with xylene and treated with ethanol at different concentrations for 5 min. Stain with hematoxylin for 5 min, treat with 5% acetic acid for 1 min, and rinse with water. Dye with eosin for 1 min and rinse with running water. Dehydrate in 70, 80, 90, and 100% ethanol for 10 s and xylene for 1 min. Drizzle with neutral gum and seal.

### TUNEL

We used the *in situ* Cell Death Detection Kit (TUNEL fluorescence FITC kit, Roche, Germany) to detect apoptotic cells. We used DAPI to stain nuclei. We used an IX73 fluorescence microscope (Olympus, Valley, PA) to analyze fluorescence staining. We used ImageJ to count the total cells and TUNEL-positive cell numbers.

### Malonaldehyde (MDA), Glutathione (GSH)/Oxidized Glutathione (GSSH) Ratio, Superoxide Dismutase (SOD), and Catalase Detection

MDA was detected using MDA detection kit (S0131S, Beyotime), GSH/GSSG ratio detection used GSH/GSSG Assay Kit (KA3779, Abnova), SOD detection used SOD assay Kit (BC0170, Solarbio), and catalase detection used Catalase Assay Kit (K773-100, Biovision). According to the protocol (Shen et al., 2020), cells were lysed, and reagents were added and the absorbance was measured with a microplate reader.

### Fluorescence *in situ* Hybridization (FISH)

The sample was grown or adhered to or sliced on the cover slide and permeated with 70% ethanol. Hybridization can be done in a traditional laboratory incubator at 37°C within 4 h. After hybridization, the washing buffer was incubated briefly to remove the excess probe. The total time is 1–1.5 h. The sample can be imaged using a standard fluorescence microscope.

### Statistical Analysis

Data were shown as mean ± SD. Student’s *t*-test or one-way ANOVA was used to compare the groups. *P* < 0.05 was considered significant.

## Results

### LncRNA TUG1 Is Upregulated in Liver Tissues After LPS Treatment

**FIGURE 1:**
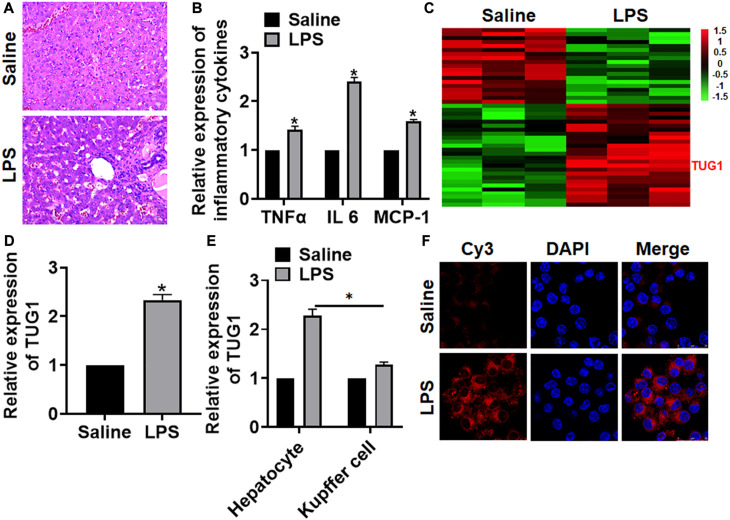
The expression of lncRNA TUG1 in LPS-induced hepatocyte inflammation. LPS (8 mg/kg) was intraperitoneally injected into mice to establish a mouse model of hepatocyte inflammation. **(A)** H&E staining for mice liver sections. Scale bar, 15 μm. **(B)** The expression of inflammatory factors TNFα, IL-6, and MCP-1 was detected by qRT-PCR. **(C)** LncRNA expression profiles in mice with saline or LPS. **(D)** The expression of TUG1 in saline- and LPS-injected livers was detected by qRT-PCR. **(E)** Hepatocytes and macrophages were separated upon saline and LPS treatment, and qRT-PCR used to test TUG1 level in hepatocyte and macrophage. **(F)** FISH assay was used to determine the location and level of TUG1 upon LPS treatment in AML12 cells. Scale bar, 25 μm. Data are mean ± SD; ^∗^*P* < 0.05. Data among multiple groups were analyzed by one-way ANOVA, followed by a Tukey *post hoc* test. The experiment was repeated in triplicate.

We first established a mouse model of hepatocyte inflammation by intraperitoneally injecting LPS, and the inflammation of liver was evaluated. LPS treatment significantly increased the infiltration of inflammatory cell (Figure 1A). Then, qRT-PCR also indicated that the expression of inflammatory factors (TNFα and IL-6) and monocyte chemoattractant protein-1 (MCP-1) was upregulated upon LPS stimulation (Figure 1B). Then, we performed bioinformatic analysis, and the data showed the differentially expressed lncRNAs in saline and LPS treatment of liver tissues, which showed an increase of TUG1 in LPS-treated liver tissues (Figure 1C). Then, qRT-PCR also indicated that TUG1 was upregulated in LPS-treated liver tissues compared with saline-treated liver tissues (Figure 1D). Considering the accumulation of macrophages (including resident and infiltrated macrophages) in liver tissue after LPS treatment, we tested the level of TUG1 in isolated hepatocytes and macrophages. qRT-PCR showed an increase of TUG1 expression in both hepatocyte and macrophage but has a higher level in hepatocyte (Figure 1E). Then, FISH assay showed that TUG1 expression, predominantly located in the cytoplasm, was dramatically raised in AML12 cells of LPS stimulation (Figure 1F).

### Knockdown of TUG1 Alleviates LPS-Induced Inflammation and Injury in Mice

**FIGURE 2:**
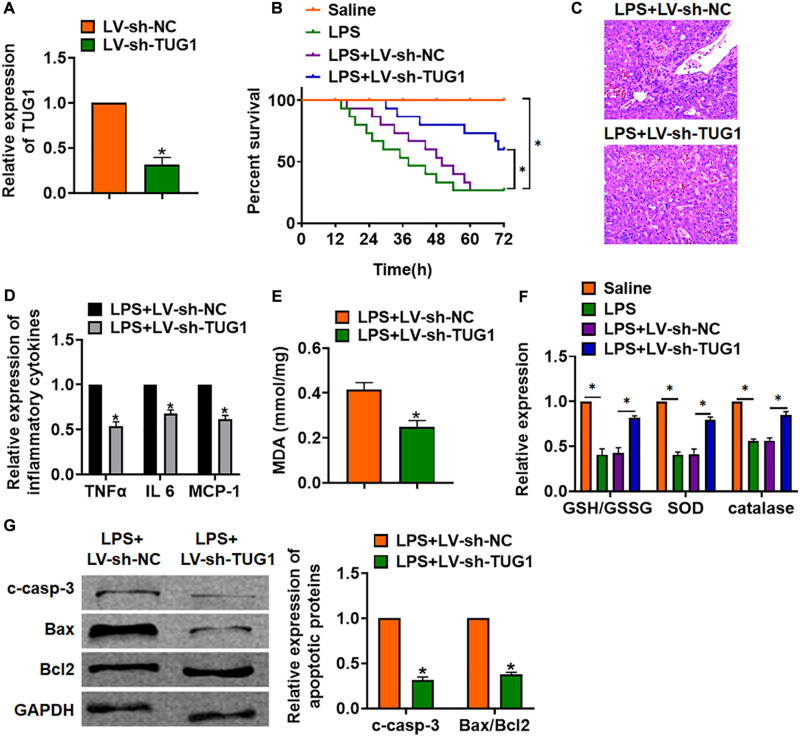
Knockdown of TUG1 alleviates LPS-induced inflammation in mice. LV-sh-TUG1 or LV-sh-NC was injected in the tail vein of mice. **(A)** The knockdown efficiency of sh-TUG1 was determined by qRT-PCR. **(B)** Survival plots for mice in different groups. **(C)** H&E staining for liver sections in different groups. Scale bar, 15 μm. **(D)** The expression of inflammatory factors TNFα, IL-6, and MCP-1 was detected by qRT-PCR. **(E)** Malonaldehyde (MDA) of livers was examined. **(F)** Glutathione (GSH)/oxidized glutathione (GSSH) ratio, superoxide dismutase (SOD), and catalase were determined. **(G)** Western blot was used to detect apoptosis-related proteins cleaved-caspase-3, Bax, and Bcl2 in mice liver tissues. Data are mean ± SD; ^∗^*P* < 0.05. Data among multiple groups were analyzed by one-way ANOVA, followed by a Tukey *post hoc* test. The experiment was repeated in triplicate.

For further research, we constructed lentiviral plasmid for knockdown of the expression of TUG1 (LV-sh-TUG1, LV-sh-NC was indicated as a control group) and injected it through the tail vein of mice (Figure 2A). The survival curve showed that LPS significantly inhibited the survival rate of mice, while deletion of TUG1 increased the survival rate compared with the LPS group (Figure 2B). H&E staining showed that LPS caused structural damage of hepatocyte and infiltration of inflammatory cells, while knockdown of TUG1 significantly alleviated the LPS-induced hepatocyte injury (Figure 2C). qRT-PCR showed that sh-TUG1 alleviated LPS-induced increase of TNFα, IL-6, and MCP-1 (Figure 2D). Then, we evaluated the oxidative stress level. MDA assay exhibited that LPS induced MDA expression, while sh-TUG1 reduced MDA level (Figure 2E). Also, silencing of TUG1 remitted LPS-induced downregulation of GSH/GSSG ratio, SOD, and catalase expression (Figure 2F). In addition, silencing of TUG1 inhibited cleaved-caspase-3 expression and suppressed Bax/Bcl2 ratio (Figure 2G).

### Deletion of TUG1 Attenuated LPS-Induced Inflammation and Injury in Cells

**FIGURE 3:**
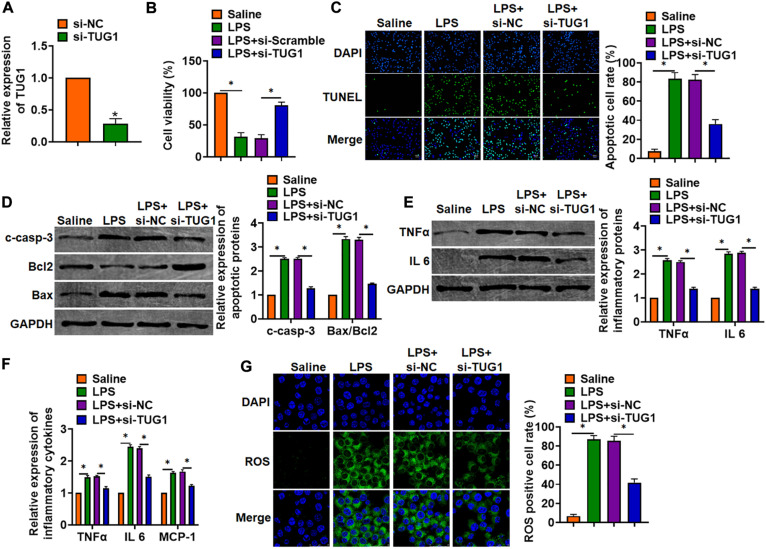
Knockdown of TUG1 attenuated LPS-induced inflammation in AML12 cells. siRNA of TUG1 was transfected into AML12 cells with LPS treatment (100 ng/ml). **(A)** The silencing efficiency of si-TUG1 was detected by qRT-PCR. **(B)** MTT assay for cell viability of AML12 cells. **(C)** Apoptosis cell numbers were tested by TUNEL staining. Scale bar, 20 μm. **(D)** Western blot for apoptosis-related proteins (cleaved-caspase-3, Bax, and Bcl2) in AML12 cells. **(E)** Western blot for TNFα and IL-6 expression. **(F)** qRT-PCR analysis for TNFα, IL-6, and MCP-1 expression. **(G)** ROS assay was performed to test the ROS level. Scale bar, 25 μm. Data are mean ± SD; ^∗^*P* < 0.05. Data among multiple groups were analyzed by one-way ANOVA, followed by a Tukey *post hoc* test. The experiment was repeated in triplicate.

*In vitro*, we cultured AML12 cells treated with LPS (100 ng/ml) to mimic *in vivo* LPS-induced hepatocyte inflammation. siRNA of TUG1 was transfected into AML12 cells to inhibit TUG1 expression (Figure 3A). MTT results showed that LPS treatment decreased cell viability, while si-TUG1 recover cell viability and remitted the injury effects of LPS (Figure 3B). In addition, TUNEL analysis exhibited an increase of apoptotic cell numbers in LPS-treated AML12 cells, while si-TUG1 decreased apoptotic cell numbers (Figure 3C). In addition, LPS promoted the expression of cleaved-caspase-3 and Bax/Bcl2, which was reversed by si-TUG1 transfection (Figure 3D). Moreover, LPS induced the expression of inflammation factors, while si-TUG1 reduced inflammation factor level (Figures 3E,F). ROS assay showed that LPS treatment promoted ROS production, while si-TUG1 inhibited ROS level in AML12 cells (Figure 3G).

### TUG1 Interacted With miR-140

**FIGURE 4:**
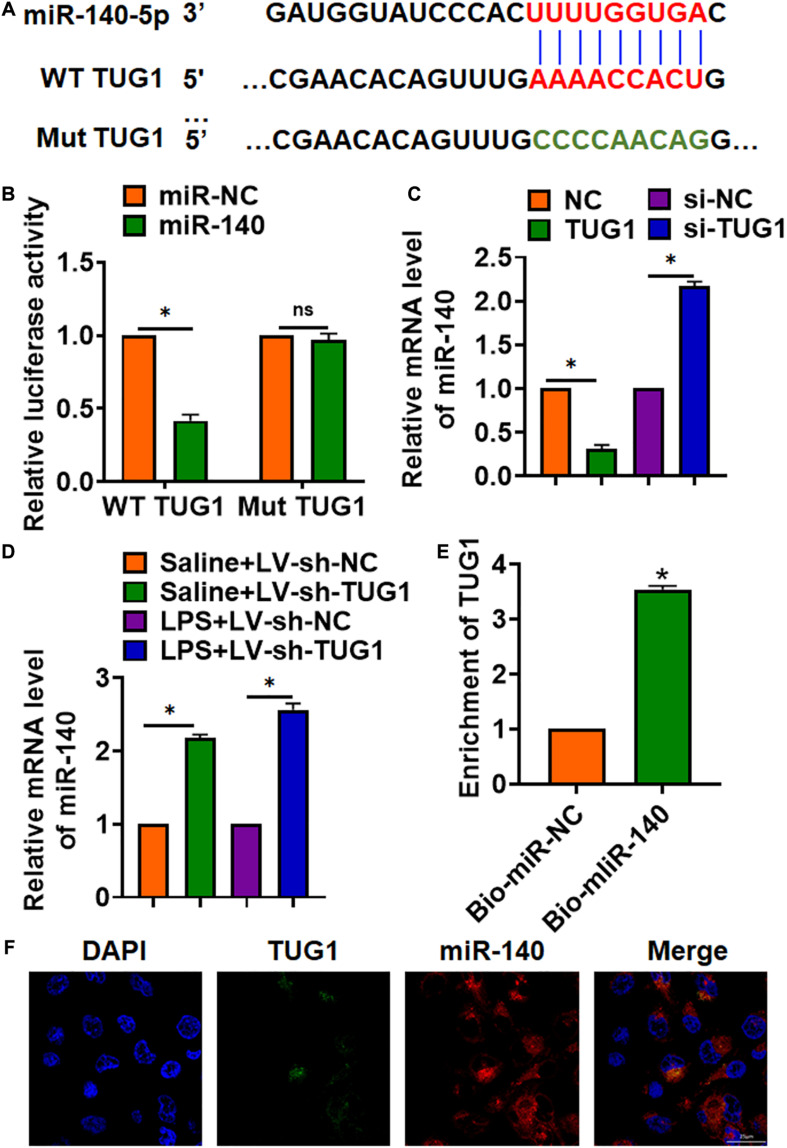
TUG1 acted as a sponge of miR-140. **(A)** MiRanda database showing the binding sites of miR-140 with TUG1, and the mutant sequence of TUG1. **(B)** Wild-type and mutant TUG1 were transfected into HEK293 cells with or without miR-140, and luciferase assay was to evaluate the binding between miR-140 and TUG1. **(C)** AML12 cells were transfected with TUG1 plasmid or si-TUG1 or its NC, and the mRNA level of miR-140 was detected using qRT-PCR. **(D)** Biotinylated miR-140 or NC was transfected into AML12 cells, and qRT-PCR was performed to detect the enrichment of TUG1. **(E,F)** FISH assay was used to determine the location TUG1 and miR-140 in AML12 cells. Scale bar, 25 μm. Data are mean ± SD; ^∗^*P* < 0.05. Data among multiple groups were analyzed by one-way ANOVA, followed by a Tukey *post hoc* test. The experiment was repeated in triplicate.

To explore the molecular mechanism of TUG1 in LPS-induced hepatocyte inflammation, we used miRanda database and found a potential binding between TUG1 and miR-140-5p (miR-140) (Figure 4A). Then, luciferase assay showed miR-140 inhibited activity of WT TUG1 not mut TUG1 in HEK293 cells (Figure 4B). Overexpression of TUG1 inhibited miR-140 level, while silencing of TUG1 promoted miR-140 level in AML12 cells (Figure 4C). Injection of LV-sh-TUG1 promoted miR-140 level with or without LPS treatment (Figure 4D). Further, endogenous TUG1 was enriched in biotinylated miR-140 transfected AML12 cells, which reveals a direct binding of TUG1 with miR-140 (Figure 4E). Then, FISH analysis showed that TUG1 was co-located with miR-140 in AML12 cytosol (Figure 4F).

### MiR-140 Inhibited TNF Expression

**FIGURE 5:**
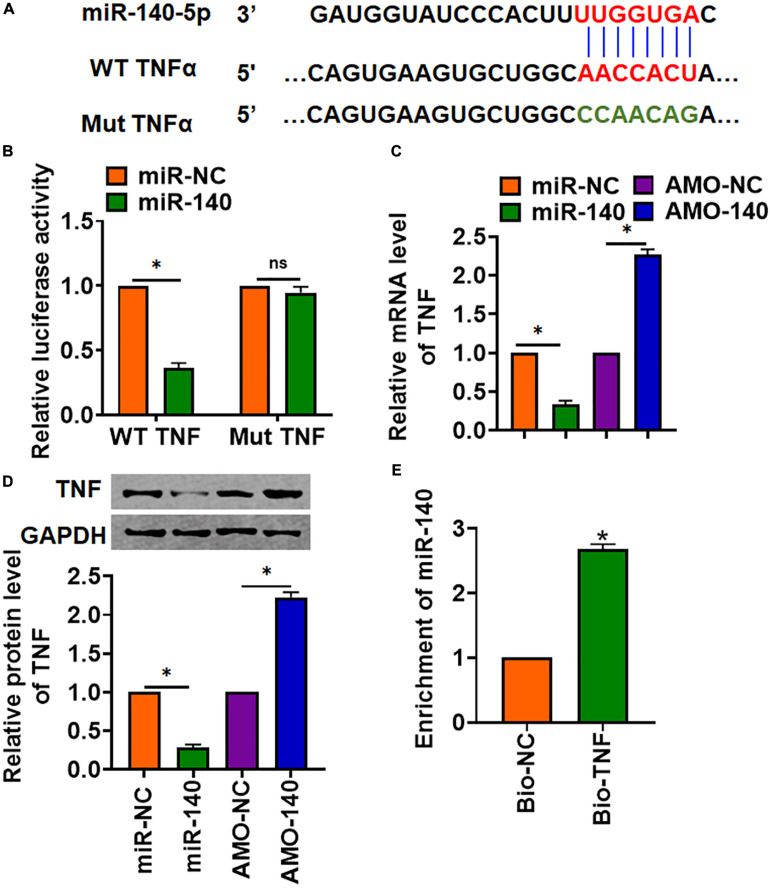
TNF was a directed target of miR-140. **(A)** The binding bases of miR-140 and TNF from Targetscan. **(B)** Wild-type and mutant TNF was transfected into HEK293 cells with or without miR-140, and luciferase assay was used to evaluate the binding. AML12 cells were transfected with miR-140 or AMO-140; **(C)** the mRNA **(D)** and protein level of TNF were detected. **(E)** RIP assay for the binding of miR-140 and TNF in AML12 cells. Data are mean ± SD; ^∗^*P* < 0.05. Data among multiple groups were analyzed by one-way ANOVA, followed by a Tukey *post hoc* test. The experiment was repeated in triplicate.

Through Targetscan, we found base pairing of miR-140 and TNFα (TNF) (Figure 5A). The following luciferase analysis suggested miR-140 directly inhibited TNF expression (Figure 5B). Furthermore, miR-140 suppressed TNF mRNA and protein expression, but AMO-140 increased TNF level in AML12 cells (Figures 5C,D). RIP assay showed enrichment of miR-140 in biotinylated TNF cells (Figure 5E).

### Deletion of TUG1 Alleviates LPS-Induced Inflammation and Injury via miR-140/TNF Axis in AML12 Cells and Liver Tissues

**FIGURE 6:**
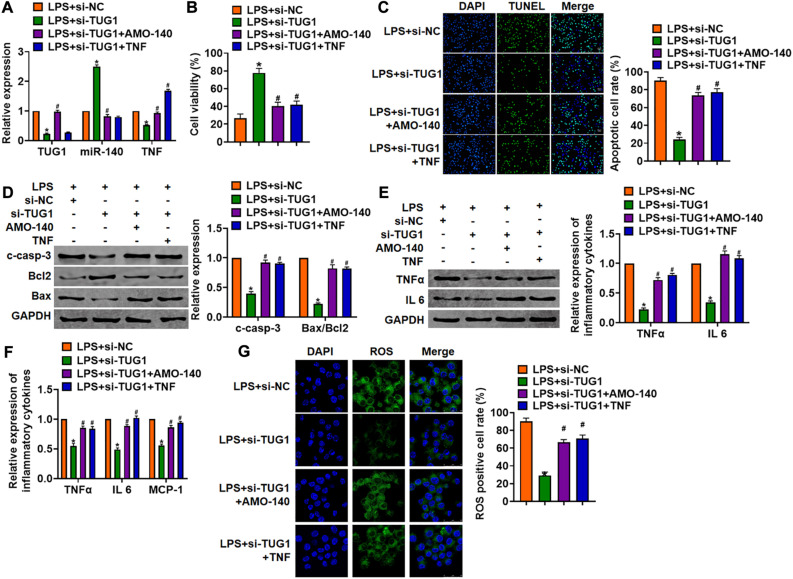
Inhibition of TUG1 alleviates LPS-induced inflammation and injury via miR-140/TNF axis in AML12 cells. Si-TUG1 was transfected into AML12 cells with AMO-140 or TNF. **(A)** The transfection efficiency was detected using qRT-PCR. **(B)** MTT assay for cell viability of AML12 cells. **(C)** Apoptosis cell numbers were tested by TUNEL staining. Scale bar, 20 μm. **(D)** Western blot for cleaved-caspase-3, Bax, and Bcl2 in AML12 cells. **(E)** qRT-PCR analysis for IL 1β, IL-6, and TNFα expression. **(F)** Western blot for TNFα and IL-6 expression. **(G)** ROS assay for the ROS level in cells. Scale bar, 25 μm. Data are mean ± SD; ^∗^*P* < 0.05. Data are mean ± SD; ^∗^*P* < 0.05 vs. LPS + si-NC, ^#^*P* < 0.05 vs. LPS + si-TUG1. Data among multiple groups were analyzed by one-way ANOVA, followed by a Tukey *post hoc* test. The experiment was repeated in triplicate.

**FIGURE 7:**
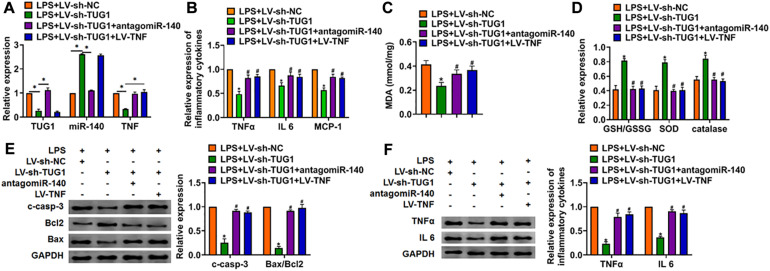
Inhibition of miR-140 or overexpression of TNF reversed the anti-inflammatory role of sh-TUG1 *in vivo*. Either LV-sh-TUG1 alone or with antagomiR-140/LV-TNF was injected in the tail vein of mice, and then LPS was injected through the tail vein. **(A)** The expression of TUG1, miR-140, and TNF in liver tissues was evaluated. **(B)** The expression of inflammatory factors TNFα, IL-6, and MCP-1 was detected by qRT-PCR. **(C)** MDA of livers was examined. **(D)** GSH/GSSH ratio, SOD, and catalase were determined. **(E)** Western blot was used to detect apoptosis-related proteins cleaved-caspase-3, Bax, and Bcl2 in mice liver tissues. **(F)** Western blot for the protein level of TNFα and IL-6. Data are mean ± SD; ^∗^*P* < 0.05. Data among multiple groups were analyzed by one-way ANOVA, followed by a Tukey *post hoc* test. The experiment was repeated in triplicate.

We then inhibited the expression of TUG1 with AMO-140 or TNF in AML12 cells (Figure 6A). Knockdown of TUG1 inhibited LPS-induced apoptosis, inflammation response, and ROS production (Figures 6B–G). However, AMO-140 or TNF removed the beneficial role of si-TUG1 on AML12 cells (Figures 6B–G). Similarly, LPS-treated mice were injected with either LV-sh-TUG1 alone or with antagomiR-140/LV-TNF (Figure 7). We also found that inhibition of miR-140 or overexpression of TNF reversed the anti-inflammatory effect of TUG1 deletion (Figures 7A–F).

## Discussion

Hepatitis is a worldwide public health problem (Martin et al., 2015). The inflammatory response caused by hepatitis severely damages liver structure and function, and 15–25% of patients with hepatitis will eventually die of liver cirrhosis or liver cancer (Tan et al., 2020). In recent years, studies have found that lncRNA regulated gene expression at the transcriptional and post-transcriptional levels, respectively. The dysfunction of these lncRNAs may lead to disease (Zhu et al., 2019). Present data showed that lncRNA TUG1 was upregulated in LPS-induced hepatocyte inflammation. Deletion of TUG1 inhibited LPS-induced inflammation response *in vivo* and *in vitro*. Furthermore, TUG1 acted as a sponge of miR-140, and miR-140 directly targeted TNF. Functionally, miR-140 or si-TNF remitted the beneficial effects of TUG1 on LPS-induced hepatocyte inflammation response.

At present, studies have shown that a variety of lncRNAs expression levels have changed significantly in liver diseases and play a core regulatory role in the occurrence, development, and prognosis of liver disease (De Vincentis et al., 2020). Therefore, lncRNA is expected to become a potential diagnostic marker, prognostic index, and clinical treatment target for hepatitis, liver cirrhosis, and liver cancer (Zhang K. et al., 2019a). Some studies have shown that lncRNA Lethe can bind to Rela and then block the binding of NF-κB to the target gene’s promoter, thus blocking the inflammatory immune response mediated by NF-κB (Rapicavoli et al., 2013). Besides, the overexpression of lncRNA CRNDE in astrocytes increased the expression of critical factors in the Toll-like receptor signal pathway, especially the signal pathway mediated by Toll-like receptor 3. Also, CRNDE also increased the expression level of downstream transcription factors, such as NF-κB and various cytokines (Li H. et al., 2017a). CRNDE regulates kidney injury by triggering inflammatory response through the TLR3-NF-κB signal pathway (Sun et al., 2019). It has also been reported that lncRNA HOTAIR is upregulated in the septic model, accompanied by the production of TNFα and the phosphorylation of p65. Knocking down HOTAIR can protect the cardiac inflammatory response and myocardial dysfunction induced by LPS (Wu et al., 2016). As a component of the outer wall of the gram-negative bacteria cell wall, LPS is commonly used to stimulate inflammation of different cells and tissues by extracellular treatment, including pneumonia (Zhang H. et al., 2020), hepatitis (Schwaderer et al., 2020), and myocarditis (Wu et al., 2016). In this study, we screened the significantly high expression of lncRNA TUG1 in the liver of mice induced by LPS. The silencing of TUG1 inhibited the LPS-induced inflammation response. Cytokines, including TNFα, IL-6, and MCP-1, were determined to assess LPS-induced inflammation. IL-6 has been considered as a pro- as well as an anti-inflammatory cytokine, and the present study indicated an increase of IL-6 upon LPS treatment. There are reports showing that IL-6 is significantly upregulated in LPS-treated liver tissues (Antoniades et al., 2014).

There is a close relationship between inflammation and oxidative stress, and they promote each other (Lee et al., 2020). It has been reported that oxidative stress is an essential liver injury mechanism caused by paracetamol (Shi et al., 2020). As one of the end products of lipid peroxidation, MDA is one of the classical indicators to reflect the degree of oxidative damage in the body (Antoniou et al., 2017). At the same time, there are various antioxidant enzymes in the liver, which can resist free radical damage, such as SOD, catalase, and GSH-Px to form an antioxidant enzyme system (Derakhshesh et al., 2019). GSH is not only a scavenger of low-molecular free radicals but also a substrate of GSH-Px, which can prevent cytokines from oxidative damage (Xuan et al., 2020). Lipid peroxidation converts ROS into active chemicals, which amplifies ROS’ effect by chain or chain branching reaction. Simultaneously, due to the accumulation of ROS, the consumption of antioxidants in liver tissue increases, and the expression of SOD, CAT, GSH-Px, and GSH decreases (Zhang R. et al., 2019b). We evaluated the role of TUG1 in LPS-induced hepatocyte inflammation by detecting oxidative stress. Our results showed that LPS treatment increased MDA level; decreased GSH/GSSG ratio, SOD, and catalase expression; and promoted ROS production. However, the knockdown of TUG1 reversed the damage of LPS. Besides oxidative stress, apoptosis is also accompanied by LPS-induced inflammation (Chen et al., 2017; Lv et al., 2020; Zhou and Xia, 2020). We further detected apoptotic marker proteins cleaved-caspase 3, Bax, and Bcl 2 in LPS-treated cells, which indicated that silencing TUG1 inhibited LPS-induced cell apoptosis.

In terms of mechanism, we found that TUG1 acted as a sponge of miR-140, and miR-140 directly targeted TNF in AML12 cells. MiR-140 was shown to modulate the angiogenesis in hepatocellular carcinoma (Hou et al., 2020). TNF is an important inflammatory factor and involved in inflammation response of multiple organs (Liu R. et al., 2019; Kaaij et al., 2020). Our data suggested that deletion of TUG1 relieved LPS-induced hepatocyte inflammation and injury by regulating miR-140/TNF axis both *in vivo* and *in vitro*.

## Conclusion

In summary, our data revealed that knockdown of TUG1 protected against LPS-induced hepatocyte inflammation and injury, which was mediated by miR-140/TNF axis. This study might provide a new understanding for the hepatitis and liver injury mechanism.

## Data Availability Statement

The original contributions presented in the study are included in the article/supplementary material, further inquiries can be directed to the corresponding author/s.

## Ethics Statement

The animal study was reviewed and approved by Linyi people’s hospital.

## Author Contributions

Q-ML, L-LL, and X-DL performed the majority of experiments, collected, and analyzed the data. PT, HX, and Z-LL performed the molecular investigations. L-KW designed and coordinated the research. Q-ML wrote the manuscript. All authors contributed to the article and approved the submitted version.

## Conflict of Interest

The authors declare that the research was conducted in the absence of any commercial or financial relationships that could be construed as a potential conflict of interest.
